# Effect of metformin on maternal and neonatal outcomes in pregnant obese non-diabetic women: A meta-analysis

**Published:** 2017-08

**Authors:** Ahmed Elmaraezy, Abdelrahman Ibrahim Abushouk, Amany Emara, Omar Elshahat, Hussien Ahmed, Magdy I. Mostafa

**Affiliations:** 1 *Faculty of Medicine, Al-Azhar University, Cairo, Egypt.*; 2 *Faculty of Medicine, Ain Shams University, Cairo, Egypt.*; 3 *Faculty of Medicine, Zagazig University, Zagazig, Egypt.*; 4 *Department of Obstetrics and Gynecology, Faculty of Medicine, Cairo University, Cairo, Egypt.*

**Keywords:** Birth weight, Diabetes mellitus, Metformin, Obesity, Pregnancy

## Abstract

**Background:**

Metformin reduces maternal and neonatal weight gain in gestational diabetes mellitus; however, this effect is poorly investigated in non-diabetic women.

**Objective:**

We performed this meta-analysis to investigate the effect of metformin intake during pregnancy on maternal and neonatal outcomes in obese non-diabetic women.

**Materials and Methods:**

We searched Medline, EMBASE, and Cochrane CENTRAL for eligible randomized controlled trials addressing the efficacy of metformin in pregnant obese non-diabetic women. Data were extracted and analyzed using RevMan software (Version 5.3). Neonatal birth weight was the key outcome. Secondary outcomes included maternal weight gain, the incidence of preeclampsia, and neonatal adverse effects (miscarriage, stillbirth and congenital anomalies).

**Results:**

Pooled data from two RCTs (n=843) showed that metformin caused a significant reduction in maternal gestational weight gain (MD-1.35, 95% CI: [2.08, -0.630]), compared to placebo. The summary effect-estimate did not favor either of the two groups in terms of reduction of neonatal birth weight Z score (MD-0.09, 95% CI: [0.23, 0.06]). Metformin was associated with 41% reduction in the risk of preeclampsia; however, this reduction was not statistically significant [RR 0.59, 95% CI: [0.03, 11.46]). None of the neonatal adverse events including stillbirth [RR 1.14, 95% CI: 0.42, 3.10]) and congenital anomalies (RR= 1.36, 95% CI: [0.58, 3.21]) differed significantly between the two groups.

**Conclusion:**

For obese pregnant women, metformin could decrease gestational weight gain with no significant reduction in neonatal birth weight. In light of the current evidence, metformin should not be used to prevent poor pregnancy outcomes in obese non-diabetic women.

## Introduction

Maternal obesity during pregnancy is a major health problem that affects 25% of pregnant women (1). It is associated with poor pregnancy outcomes for the mother, such as increasing the risk for preeclampsia, maternal hemorrhage, and the need for caesarian section (CS) (2-6). Moreover, it is linked to poor fetal outcomes, such as fetal macrosomia, stillbirth, and increased risk of infant mortality (7). Epidemiological studies highlighted the correlation between high birth weight and the risk for adult obesity later in life (8-10). The cycle between maternal and offspring obesity contributes to the rising global prevalence of obesity and interrupting it through an effective intervention during pregnancy would delineate its burden (11). It is estimated that overweight pregnant women receive an antenatal care that is 5.4-16.2 times more expensive than women of normal weight (12). 

Former studies have shown that following dietary modifications and lifestyle interventions did not significantly reduce obesity-related complications during pregnancy (13-15). Recently, hyperglycemia and insulin resistance have been incriminated in the pathogenesis of gestational weight gain and fetal macrosomia (16, 17). These findings formulated the basis for using metformin as a prophylactic treatment in obese pregnant women to reduce the incidence of poor pregnancy outcomes. Moreover, metformin use during pregnancy has not been linked to increased birth defects in neonates (18). Previous clinical trials have shown that metformin reduces maternal weight gain in women with gestational diabetes mellitus (DM) (19, 20). 

Other studies examined the effect of metformin on pregnancy outcomes in women with polycystic ovarian syndrome (21, 22). Recently, two large randomized controlled trials (RCTs) have examined the value of metformin use during pregnancy in obese non-diabetic women. In the MOP (Metformin in obese no diabetic pregnant women) trial, metformin significantly decreased maternal weight gain during pregnancy without affecting the neonatal birth weight (23). However, in the EMPOWaR (Effect of metformin on maternal and fetal outcomes in obese pregnant women) trial, metformin was proven ineffective in reducing both outcomes (11). 

In light of these controversial findings, we performed a pooled analysis of both RCTs to evaluate the effect of metformin on maternal and fetal outcomes in obese non-diabetic women.

## Material and methods

We followed the PRISMA statement guidelines during the preparation of this meta-analysis. Moreover, all steps were performed in a strict accordance with the Cochrane handbook for systematic reviews of interventions (updated March 2011) (24).

**Literature search strategy**

We searched Medline (through PubMed), EMBASE, and the Cochrane Central Register of Controlled Trials to identify relevant studies published up to April 2016. The following search terms were used: ((Metformin) AND (Obese OR Overweight) AND (Pregnant OR Pregnancy OR Gestation*) AND (Non-diabetic OR Without diabetes)). Moreover, we scanned the reference lists of retrieved articles and checked the clinical trial registry (clinicaltrials.gov) for additional studies.

**Criteria for considering studies for this review**

Studies were eligible if they were controlled clinical trials, which compared metformin to placebo in obese, pregnant, non-diabetic women. Only studies reporting the results of infant birth weight, maternal weight gain, or birth consequences were included. 

Excluded studies comprised pharmacokinetic analysis of metformin during pregnancy, reviews, secondary data analysis, and studies with unspecified data collection and analysis methods. Three authors (AE, AE, and OE) independently applied the selection criteria. The abstract screening was performed first, and then the full-text articles of eligible abstracts were retrieved and screened for eligibility to meta-analysis.

**Data extraction**

The following data were extracted from each study by three independent authors (AE, HA, and AE): a) Study characteristics, including first author's name, publication year, sample size, mean age, dosage, and duration of metformin intake; b) The primary outcome measure was the neonatal birth-weight Z score, which was defined as the difference between observed and expected birth weight with adjustment for gestational age, divided by the fitted standard deviation (SD); c) Maternal secondary outcome measures included gestational weight gain, defined as the difference in maternal weight between the day of randomization and the last antenatal visit; d) Maternal adverse events including preterm birth before 37 wk of gestation, gestational DM, preeclampsia, pregnancy-induced hypertension, delivery by CS, postpartum hemorrhage, defined as blood loss of 1 L or more, and e) Neonatal adverse events including death before 24 wk of gestation, stillbirth at 24 wk of gestation or later, congenital anomalies, and neonatal death. When the mean and SD were not provided, we calculated them from the median and inter-quartile range according to Wan *et al* (25). 

**Risk of bias assessment in included studies**

The risk of bias in retrieved RCTs was assessed according to the Cochrane handbook of systematic reviews of interventions 5.1.0 (updated March 2011). We used the risk of bias assessment table provided in (part 2, Chapter 8.5) of the same book (26). According to Egger and colleagues, publication bias assessment is not reliable for less than 10 pooled studies (27, 28). Therefore, in the present study, we could not assess the existence of publication bias by Egger’s test for funnel plot asymmetry.

**Data synthesis**

We used Review Manager (RevMan) software (version 5.3 for windows) during data synthesis. Changes in the primary and secondary outcomes were pooled as mean difference (MD) in a fixed effect meta-analysis model, using the inverse variance (IV) method. Adverse events were pooled as risk ratios (RR) in a fixed effect model using Mantel-Haenszel (M-H) method. The existence of heterogeneity was assessed by Chi-Square test and measured by I-square test. In the case of a significant heterogeneity (Chi-Square p<0.1), the random effects model was used.

## Results

**Literature search results**

**Figure 1:**
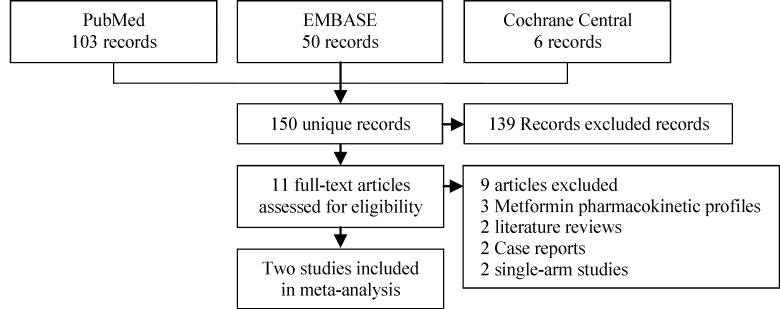
PRISMA flow diagram of studies’ screening and selection

Our search retrieved 150 unique articles. Following the abstract screening, only 11 references were eligible for full-text screening. Nine full-text articles were excluded as follows: Two single arm/irrelevant trials, three pharmacokinetic analyses, two literature reviews, and two case reports (29-37). Finally, two RCTs were found to be eligible for the final analysis (PRISMA flow diagram; Figure 1) (11, 23). The two studies included 843 pregnant women who had a body mass index (BMI) more than 30 kg/m^2^ and normal glucose tolerance. The summary of included studies and their main results are shown in table I and baseline characteristics of their patients are shown in table II.

**Risk of bias in included studies**

**Figure 2.**
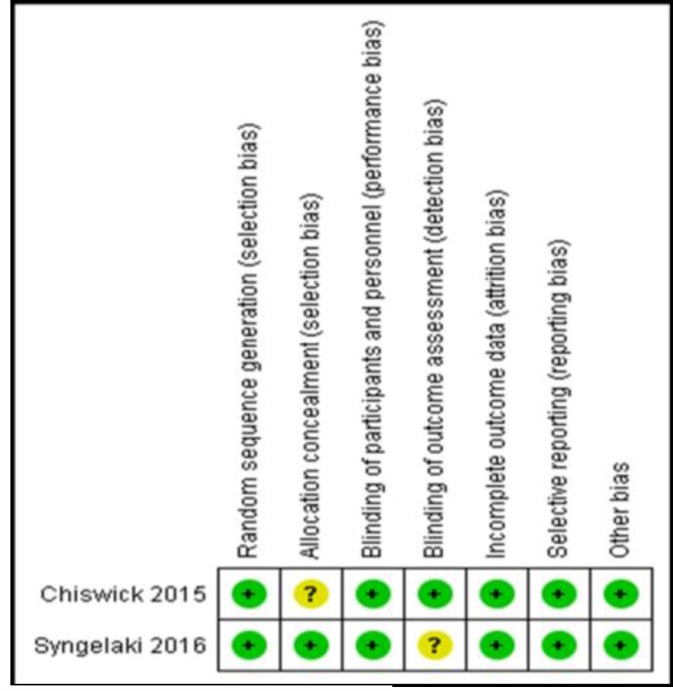
Cochrane risk of bias assessment results for included studies, showing a low risk of bias in included studies

Both included studies were of a low risk of bias in all domains, except for allocation concealment (unclear in the study by Chiswick *et al* 2015) and blinding of outcome assessors (unclear in the study by Syngelaki *et al* 2016). The summary of the risk of bias assessment domains is shown in Figure 2. Authors’ judgments with justifications are shown in Supplementary 1.

**Fetal efficacy**

**Figure 3:**
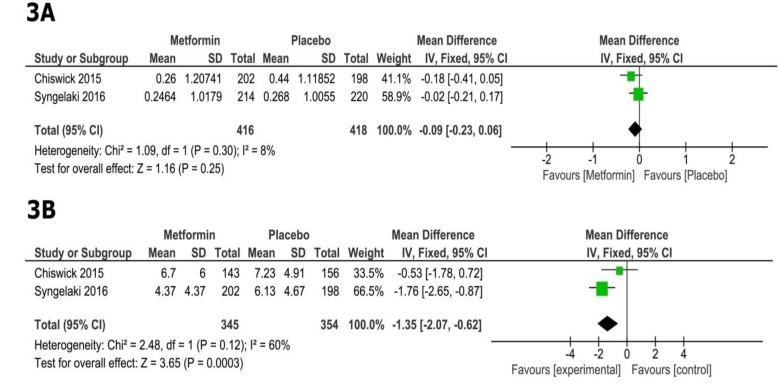
Forest plots of mean difference in A) change in neonatal birth weight Z score, and B) maternal weight gain (11, 23).

In comparison with placebo, metformin did not show a significant reduction of neonatal birth weight Z score (MD=-0.09, 95% CI [-0.23, 0.06], p=0.25; figure 3A). Pooled studies were homogenous (p=0.30, I^2^=8%).

**Maternal efficacy**

The overall MD favored the metformin group over the placebo group in terms of maternal gestational weight gain (MD= -1.35, 95% CI [-2.07, -0.62], p=0.0003; Figure 3B). Pooled studies were homogenous (p=0.12, I^2^=60%).

**Maternal safety**

**Figure 4:**
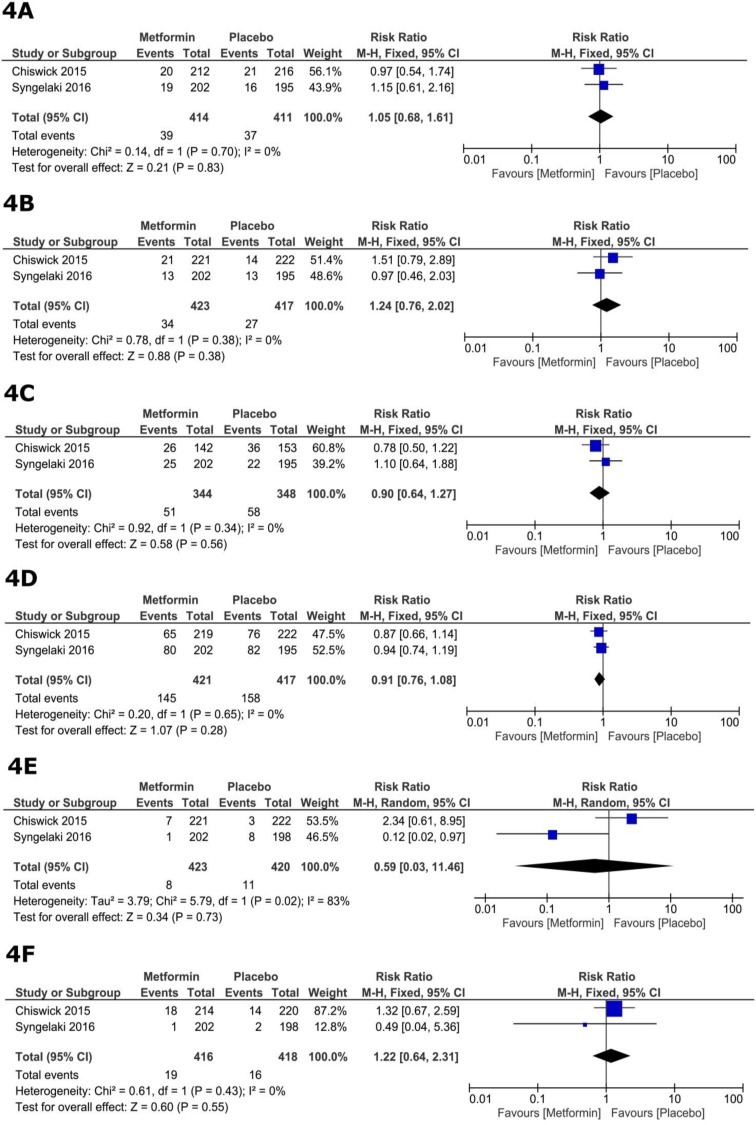
Forest plots of risk ratios of maternal adverse events.

The total number of reported maternal adverse events did not differ significantly between the metformin and placebo groups (RR=0.95, 95% CI [0.79, 1.14], p=0.59). The pooled RRs for individual adverse events were as follows: Postpartum hemorrhage (RR=1.05, 95% CI [0.68, 1.61], p=0.83; figure 4A), pregnancy-induced hypertension (RR=1.24, 95% CI [0.76, 2.02], p=0.38; figure 4B), gestational DM (RR=0.90, 95% CI [0.64, 1.27], p=0.56; figure 4C), CS (RR=0.91, 95% CI [0.76, 1.08], p=0.28; figure 4D), preeclampsia (RR=0.59, 95% CI [0.03, 11.46], p=0.48; figure 4E), and spontaneous early preterm birth (RR=1.22, 95% CI [0.64, 2.31], p=0.55; figure 4F). For all adverse events, pooled studies were homogeneous (Chi square p>0.1), except for preeclampsia (p=0.02, I^2^=83%).

**Fetal safety**

**Figure 5:**
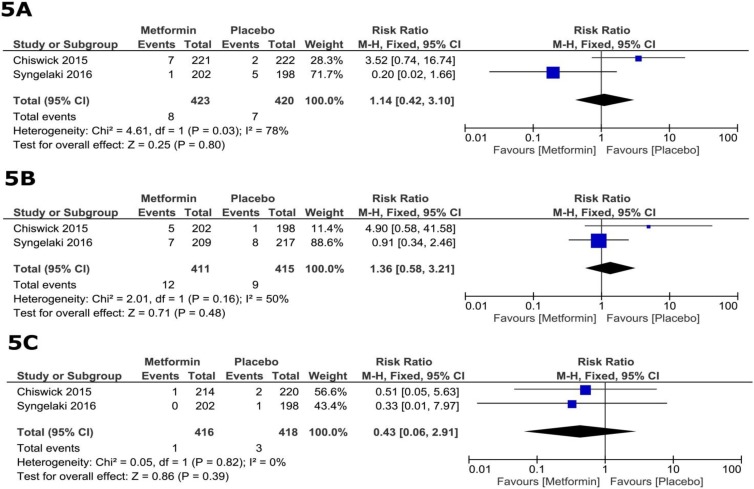
Forest plots of risk ratio of fetal adverse events (11, 23).

The total number of reported fetal adverse events did not differ significantly between the metformin and placebo groups (RR=1.30, 95% CI [0.83, 2.05], p=0.25). The pooled RRs for individual adverse events were as follows: fetal death in terms of miscarriage and stillbirth (RR=1.14, 95% CI [0.42, 3.10], p=0.80; figure 5a), congenital anomalies (RR=1.36, 95% CI [0.58, 3.21], p=0.48; figure 5b), and neonatal death (RR=0.43, 95% CI [0.06, 2.91], p=0.39; Figure 5c). 

For all adverse events, pooled studies were homogeneous (Chi square p>0.1). To account for between-study variability, we re-conducted the analysis of all fixed-effect outcomes under the random-effects model with no recorded difference in our results (Supplementary 2).

**Table I:** Shows a summary of the design and main findings of included studies

**Study ID**	**Study design**			**Intervention group**	**Population**	**Sample size**		**Main findings**
	**Design**	**Crossover or parallel**	**Blinding**			**Initial**	**ITT**	
Syngelaki 2015	Randomized, placebo-controlled trial	Parallel	Double blinded	Metformin 500-2500 mg/day	- Pregnant women aged ≥16 yr - between 12 and 16 wk gestation - BMI of 30 kg/m^2^ or more - Normal glucose tolerance	449	443	Both groups were comparable in terms of median birth weight Z score, maternal weight gain and adverse events.
Chiswick 2016	Randomized, placebo-controlled trial	Parallel	Double blinded	Metformin 3 grams/day		450	400	Metformin reduced maternal weight gain; however, there were no significant differences in birth weight Z score or birth consequences.

**Table II:** Shows baseline characteristics of enrolled women in both trials

**Study ID** ** group**		**Age (Years)**	**BMI (Kg/m** ^2^ **)**	**Gestational age**	**Race N (%)**					**Comorbidities N (%)**	
					**White**	**Black**	**Mixed**	**S Asian**	**E Asian**	**Hypertension**	**Preeclampsia**
Syngelaki 2015											
	Metformin	29.8 (5.6)	37.8 (4.7)	100 (7.9) ^a^	101 (92.7%)	3 (2.8%)	2 (1.8%)	2 (1.8%)	0 (0%)	1 (0.9%)	6 (5.5%)
	Placebo	29.6 (5)	37.5 (5.5)	98.9 (9.0) a	114 (96.6%)	2 (1.7%)	1 (0.8%)	0 (0%)	0 (0%)	1 (0.8%)	3 (2.5%)
Chiswick 2016											
	Metformin	32.9* (27.3, 36.2)	38.6* (36.5, 41.5)	15.1* (13.7, 17.0) b	142 (70.3%)	50 (24.8%)	2 (1.0%)	7 (3.5%)	1 (0.5%)	13 (6.45%)	14 (6.9%)
	Placebo	30.8* (26.6, 34.4)	38.4* (36.3, 41.9)	14.9* (13.6, 17.3) b	128 (64.6%)	55 (27.8%)	3 (1.5%)	12 (6.1%)	0 (0%)	17 (8.6%)	13 (6.6%)

Data are: mean (SD),

BMI: body mass index

S Asian: South Asian

E Asian: East Asian

* = median (IQR), or N (%)

a : days

b :week

**Supplementary 1:** Risk of bias assessment for included studies

		**Risk of Bias**	**Quotations**
Syngelaki 2016			
	Random sequence generation (Selection bias)	Low	"Eligible women were randomly assigned, in a 1:1 ratio, with the use of computer-generated random numbers, to receive either metformin or placebo."
	Allocation concealment (Selection bias)	Low	"The appearance, size, weight, and taste of the placebo tablets were identical to those of the metformin tablets; both were purchased at full cost from University College London Hospitals NHS Foundation Trust."
	Blinding of participants and personnel (Performance bias)	Low	"Double-blind"
	Blinding of outcome assessment (Detection bias)	Unclear	
	Incomplete outcome data (Attrition bias)	Low	"The analysis was performed according to the intention-to-treat principle"
	Selective reporting (Reporting bias)	Low	All outcomes were reported in a pre-specified protocol.
	Other bias	Low	No other sources of bias could be detected.
Chiswick 2015			
	Random sequence generation (Selection bias)	Low	"We randomly assigned participants (1:1), via a web based computer generated block randomization procedure."
	Allocation concealment (Selection bias)	Unclear	
	Blinding of participants and personnel (Performance bias)	Low	"Double-blind"
	Blinding of outcome assessment (Detection bias)	Low	"Members of the independent Data Monitoring Committee had access to unmasked data reports, but had no contact with study participants."
	Incomplete outcome data (Attrition bias)	Low	"All randomly assigned patients to enter safety and efficacy analysis (ITT)"
	Selective reporting (Reporting bias)	Low	All outcomes were reported in a pre-specified protocol.
	Other bias	Low	No other sources of bias could be detected.

**Supplementary 2:** Meta-analysis results under the random-effects model

**Outcome**			**Effect Estimate**	**95% CI**	**p-value**
Maternal weight gain			MD -1.23	[-2.43, -0.03]	0.04
Neonatal Birthweight Z Score			MD -0.09	[-0.024, 0.07]	0.26
Maternal adverse events					
	Preterm birth		RR 1.23	[0.64, 2.35]	0.53
	Postpartum Hemorrhage		RR 1.05	[0.68, 1.61]	0.83
	Caesarian section		RR 0.91	[0.76, 1.09]	0.30
	Pregnancy-Induced Hypertension		RR 1.24	[0.76, 2.03]	0.38
	Gestational Diabetes Mellitus		RR 0.90	[0.63, 1.27]	0.53
Fetal adverse events					
	Stillbirth		RR 0.91	[0.05, 15.55]	0.95
	Congenital anomalies		RR 1.61	[0.33, 7.87]	0.56
	Neonatal Death		RR 0.44	[0.06, 2.97]	0.40

CI: Confidence Interval

MD: Mean Difference

RR: Risk Ratio

## Discussion

The results of our analysis indicate that using metformin in obese, non-diabetic pregnant women significantly reduced maternal weight gain; however, it was not associated with a significant reduction in neonatal birth weight. The incidence of gestational DM, preeclampsia, as well as other maternal and fetal adverse events, did not differ significantly between the metformin and placebo groups. Our analysis supports the results of the MOP trial regarding the effect of metformin on maternal weight gain. This can be explained by the fact that the MOP trial was designed to address the limitations of the former EMPOWaR trial by increasing adherence to treatment and using a higher metformin dose of 3 grams per day instead of 2.5 gr/day in the EMPOWaR study. Observational studies have also shown that metformin can significantly reduce maternal weight gain in women with gestational DM or polycystic ovarian syndrome (19, 20, 38).

On the other hand, our results are in agreement with the EMPOWaR trial regarding the lack of metformin efficacy in reducing the incidence of preeclampsia. Although metformin reduced the levels of interleukin-6 (IL-6) and C-reactive protein (CRP), which are commonly elevated in preeclampsia cases, the incidence of pre-eclampsia was similar in both arms of the EMPOWaR study (11). Similarly, other studies have shown that metformin intake during pregnancy does not decrease the risk of preeclampsia or gestational DM (21, 39). These findings challenge the hypothesis of causality or association between elevated levels of IL-6 and CRP inflammatory markers with the incidence and severity of preeclampsia or preterm birth (40-42).

Former pharmacological analyses have shown that metformin has a similar pharmacokinetic profile in both pregnant and non-pregnant women with the ability to cross the placenta, reaching similar concentrations in the fetal plasma to those in the maternal circulation (43). Regarding its pharmacodynamic effect, it was found to effectively reduce insulin secretion and insulin resistance during pregnancy in both randomized and longitudinal studies (38, 44). The EMPOWaR trial reported a lack of effect, manifested by elevated glucose levels at 36 weeks of gestation, indicating possible homeostatic changes in glucose metabolism during pregnancy (11). The safety of metformin has been investigated before (18). Our pooled analysis aimed to provide an adequate sample size to further investigate the association between metformin intake in pregnancy and poor gestational outcomes as fetal death or congenital anomalies. Our results show that metformin does not increase the risk for adverse maternal or fetal outcomes.

Although metformin conducted its pharmacodynamic effect (lowering blood glucose and insulin concentrations) in enrolled patients of both studies, it did not significantly affect the neonatal birth weight. As confirmed by the EMPOWaR trial, these results present a challenge to the 1952 Pederson theory that states that maternal hyperglycemia stimulates fetal hyperinsulinemia and therefore, increasing fetal weight (45). Other factors that can link obesity and fetal macrosomia as elevated blood lipids or disturbed levels of adipokines in obese women should be investigated (46, 47).

Despite the lack of effect on neonatal birth weight, follow up of these siblings should be carried out to track the effect of metformin in their adult life. A former animal study showed that prenatal intake of metformin reduced the risk of obesity and glucose intolerance during adulthood (48). Moreover, a clinical trial on metformin in women with gestational DM showed that children, born to mothers who received metformin during pregnancy, had lower visceral fat at two years of age than children, born to mothers with insulin therapy during pregnancy (20). The mechanism of how metformin affects body weight during the adult life of the offspring should be further investigated.

Strength points: The large sample size of the included studies adds to the power of our analysis and increases its potential for generalizability. Moreover, including a widely heterogenous population in the MOP trial may allow for generalizing the results of this analysis beyond the studied population.

**Limitation**

Limitations for pooling data to summarize few studies include selective reporting, inadequate accounting for heterogeneity and publication bias. The fact that the MOP trial used a higher metformin dose on a more diverse population than the EMPOWaR trial may serve as another limitation. Further studies are warranted to verify the impact of metformin on both maternal and neonatal weights. Although not significant, few adverse events, such as preeclampsia, need for caesarian section, and neonatal death had a trend towards increasing in the metformin group; therefore, future studies are needed to further assess these outcomes. We are aware of another ongoing trial on the subject (ACTRN12612001277831).

**Recommendations**

Despite the absence of major complications in the metformin groups, minor adverse events as gastrointestinal dysregulation, commonly seen with metformin, limit participation in these clinical trials. Finding ways to improve the pharmacokinetic profile of metformin can effectively ameliorate these side effects and increase patients' participation in future studies. As stated earlier, the safety and efficacy of metformin should be followed-up in both mothers and siblings to evaluate the long term results of metformin use in pregnancy.

## Conclusion

To recapitulate, metformin intake during pregnancy in obese, non-diabetic pregnant women reduces maternal weight gain without significant reduction of neonatal birth weight. In light of the current evidence, metformin should not be used to prevent poor pregnancy outcomes in obese non-diabetic women.

## Conflict of interest

The authors have no conflicts of interest to declare.
